# Enhanced tetracycline degradation with TiO_2_/natural pyrite S-scheme photocatalyst

**DOI:** 10.1038/s41598-024-54549-0

**Published:** 2024-02-29

**Authors:** Masoumeh Hasham Firooz, Azra Naderi, Masoud Moradi, Roshanak Rezaei Kalantary

**Affiliations:** 1 Department of Environmental Health Engineering, School of Public Health, Tehran University of Medical Sciences, Tehran, Iran; 2 Department of Environmental Health Engineering, School of Public Health, Iran University of Medical Sciences, Tehran, Iran; 3 Research Center for Environmental Health Technology (RCEHT), Iran University of Medical Sciences, Tehran, Iran; 4 Research Center for Environmental Determinants of Health (RCEDH), Health Institute, Kermanshah University of Medical Sciences, Kermanshah, Iran

**Keywords:** Photocatalytic removal, TiO_2_/NP, TiO_2_, Pyrite, Tetracycline (TC), Pharmaceutical wastewater, Environmental chemistry, Chemical engineering, Catalysis, Catalyst synthesis, Photocatalysis

## Abstract

In this study, TiO_2_ nanoparticles were employed as a photocatalyst for the degradation of tetracycline (TC) under visible light irradiation. The TiO_2_ nanoparticles were decorated on natural pyrite (TiO_2_/NP) and characterized using XRD, FTIR, and SEM–EDX methods. This study evaluated the impacts of various operational parameters such as pH, catalyst dosage, initial TC concentration, and light intensity on TC removal. The findings revealed that under optimal conditions (pH 7, catalyst: 2 g/L, TC: 30 mg/L, and light intensity: 60 mW/cm^2^), 100% of TC and 84% of TOC were removed within 180 min. The kinetics of TC elimination followed a first-order model. The dominant oxidation species involved in the photocatalytic elimination of TC was found to be ^·^OH radicals in the TiO_2_/NP system. The reuse experiments showed the high capability of the catalyst after four consecutive cycles. This study confirmed that the TiO_2_/NP system has high performance in photocatalytic TC removal under optimized experimental conditions.

## Introduction

Pharmaceutical compounds (PCs) are known to be potential contaminants of water resources due to their extensive use in medical purposes, which results in the release of high concentrations of these compounds into pharmaceutical and hospital wastewater^1^.

Numerous studies have been interested in the occurrence of pharmaceutical compounds as emerging pollutants in water resources owing to the threats these compounds at low concentrations pose to the ecosystem, human health, and water resource quality^2,3^.

Antibiotics are one of the pharmaceutical compounds categories which are discharged into water matrices from pharmaceutical factories, hospitals, municipal sewages, and livestock farms^4^. There may be major environmental problems due to the rising production and excessive usage of these substances, which may be hazardous to both humans and animals^5,6^. The majority of them are non-biodegradable, which means that they can remain in the ecosystem for extensive periods of time and generate antibiotic resistance bacteria^7^. Among these compounds, tetracycline (TC) has been found in wastwater effluent and in trace amounts in surface water^8–11^. Since TC is resistant to microbial degradation, biological wastewater treatment will not be able to complete the removal of this pollutant, and it can be considered a challenge for wastewater treatment systems^12^. As a result, because of its adverse impacts, the development of sustainable technologies is essential to eliminate this pollutant from environmental aqueous.

TC has been eliminated from liquid solutions using a variety of methods, including biological, chemical, and physical processes^11,13–15^. However, most of these processes are expensive or unsuitable for effectively removing TC. Because of its high success in eliminating refractory organic pollutants from wastewater, advanced oxidation processes (AOPs) have gained significant attention in recent years^16,17^. Among these methods, photocatalytic oxidation stands out as a particularly intriguing choice for reducing contaminants. The basis of this process is the employment of semiconductors in the presence of light, and it is highly effective, straightforward, fast, and has minimal toxicity^18–21^. The photocatalytic features of various semiconductors in the elimination of various antibiotics are presented in Table 1.

**Table 1:** Different photocatalysts applied for antibiotic degradation.

No	Catalyst	Light source	Experimental conditions	Antibiotic	Efficiency (%)	Ref
1	CZ@T-GCN	Simulated sunlight (250 W xenon)	[C_0_]: 60 mg/L, [catalyst]: 0.9 g/L, [pH]:7, [time]:120 min	Amoxicillin	100	^4^
2	Cu-TiO_2_ /GO	UV light (300 W mercury lamp)	[C_0_]: 20 mg/L, [catalyst]: 0.5 g/L, [pH]:7, [time]:90 min	Tetracycline hydrochloride	98	^38^
3	TiO_2_	Visible light	[C_0_]: 10 mg/L , [catalyst]: 0.2 g/L, [temperature]:25 °C, [time]:120 min	Tetracycline	56.7	^23^
4	ZnO@BiOBr	Visible light	[C_0_]: 20 ppm , [catalyst]: 0.5 g/L, [pH]:6, [time]:112 min	Metronidazole	87.72	^39^
5	CdS/BiOBr	Visible light	[C_0_]: 10 mg/L, [catalyst]: 50 mg, [pH]:7, [time]:240 min	Norfloxacin	98.3	^40^
				ciprofloxacin	96.7	
6	Ti-MOF/Ag/NiFeLDH	Visible light (300 W xenon)	[C_0_]: 15 mg/L, [catalyst]: 10 mg, [time]:70 min	Levofloxacin	92	^19^
7	BiVO_4_	Sunlight	[C_0_]: 10 mg/L, [catalyst]: 50 mg, [time]:240 min	Oxytetracycline	83	^41^
		visible light			55.5	
8	Ag-ZnO	UV light	[C_0_]: 10 mg/L, [catalyst]: 50 mg, [time]:180 min	Ofloxacin	100	^42^
		Sunlight			100	
9	Zn_1−x_Mg_x_Fe_2_O_4_	Visible light (300 W xenon)	[C_0_]: 10 mg/L, [catalyst]: 0.3 mg/ml, [temperature]:30 ± 0.5℃, [pH]:7, [time]:90 min	Sulfadiazine	99.1	^43^
10	Urea/TiO_2_/ZnFe_2_O_4_/zeolite	UV–visible light	[C_0_]: 100 mg/L, [catalyst]:2 g/L, [pH]:7, [time]:120 min	Cephalexin	74	^44^
			[C_0_]: 100 mg/L, [catalyst]:2 g/L, [pH]:5, [time]:120 min	Metronidazole	70	
11	Ta_3_N_5_/CdS	Visible light (300 W xenon)	[C_0_]: 20 mg/L, [catalyst]: 30 mg, [time]: 50 min	Tetracycline	90.5	^45^
12	MIL-101(Fe)/BiOBr	Visible light (300 W xenon)	[C_0_]: 10 mg/L, [catalyst]:25 mg, [pH]:6.8, [time]: 40 min	Enrofloxacin	84.4	^46^
13	MIL-101(Fe)/Bi_2_WO_6_	Visible light	[C_0_]: 20 mg/L, [catalyst]:20 mg, [pH]: 5.2, [time]: 60 min	Tetracycline	82.8	^47^
14	ZnO/g-C_3_N_4_	Visible light (fluorescent bulb)	[C_0_]: 1 mg/L, [catalyst]:0.05 g/L, [pH]: 8, [time]: 120 min	Ciprofloxacin	93.8	^48^

Among them, titanium dioxide (TiO_2_) is one of the greatest broadly used catalysts because of its outstanding performance, chemical resistance, availability, low toxicity, and inexpensive cost^22,23^. Nevertheless, the inherent characteristics of pure TiO_2_, namely its substantial band gap and swift recombination rate of electron–hole pairs generated, hinder proper photocatalyst efficiency under visible light^19,22^. Additionally, in practical uses, since nanoparticles are applied as suspension, separating and recovering nanoparticles from treated aqueous solution is a serious problem^24^. Therefore, the catalytic activity of TiO_2_ can be increased through transition metals doping and substrates immobilizing approaches^25–27^. In other hands, one of the typical strategy for improving photocatalyst performance is to create heterogeneous catalysts that provide effective spatial separation of photocarriers. In particular, S-scheme photoscatalysts with a strong internal electric field (IEF) demonstrate considerable advantages in photocarrier separation efficiency and photoreduction capabilities^28–31^. These improved S-scheme catalysts still have issues such as easy compaction and difficult separation and recovery, which may lead to secondary contamination, limiting their industrial applicability^32,33^.

Natural semiconductor minerals (NSMs) have been utilized to boost the photocatalytic efficacy when irritated by visible light due to their impurities and complex crystal imperfections. In addition, their low cost and availability can significantly reduce treatment costs^34^. Natural pyrite (NP), also known as FeS_2_, is a readily accessible non-semiconductor material (NSM) that possesses a reasonably high potential for optical absorption due to its modest band gap of 0.95 eV. This makes it a promising candidate for modifying TiO_2_, offering a practical alternative^35^. As a result, immobilizing nanoparticles on pyrite structures can be an excellent approach to stabilize nanoparticles and provide higher photocatalytic contact surface area, which promotes photocatalytic activity.

Recently, S-scheme heterojunction photocatalysts have attracted the attention of many researchers. In this approach, due to the heterojunction of the S-scheme, the separation rate of photogenerated electrons and holes improved, and at the same time, the redox potential does not decrease and conductivity is also preserved^36^. In S-scheme photocatalysts, electrons move from the conduction band of one semiconductor to the valence band of the other semiconductor due to the internal electric field at the interface between the two semiconductors. This creates an S- shaped electron migration path. Therefore, electrons and holes that have a strong redox potential are preserved, and those with a weaker strong redox potential are transferred and consumed^37^.

Given the foregoing, the principle aim of the current study is to assess the effectiveness of natural pyrite supported TiO_2_ (TiO_2_/NP) in TC elimination using the visible light. Additionally, the impact of various factors, including pH, catalyst quantity, initial TC concentration, light intensity, the existence of mineral ions on the photocatalytic removal of tetracycline, as well as mineralization degree by TiO_2_/NP, was evaluated.

## Materials and methods

### Chemicals

The tetracycline powder, obtained from Sigma-Aldrich Co. (USA), with a purity of at least 88% and meeting HPLC grade standards, was used in the study. The chemical properties of tetracycline are detailed in Table 2. The pyrite mineral used in this study was obtained from the Department of Mine Engineering at Tehran University in Iran. All other chemicals, including tert-butyl alcohol (TBA), potassium iodide (KI), oxalic acid, nitric acid, ethanol, and tert-butyl titanate (TBT), were acquired from Merck Company (Germany) and utilized without any further purification. Deionized water (DIW) was utilized in the creation of the watery mixtures.

**Table 2:** Characteristics of tetracycline powder.

Chemical structure	Molecular formula	M_w_ (g/mol)	solubility	PK_a1_	PK_a2_	PK_a3_
	C_22_H_24_N_2_O_8_.xH_2_O	444.43	1.7 g/L	3.30	7.68	9.69

### Synthesis of TiO_2_/NP

The pyrite stone was machine-crushed and milled, then ultrasonicated for 5 min in ethanol (95%). After being washed in HNO_3_ (1 M) to remove external contaminants, the resulting pyrite was rinsed multiple times in deionized water and dried at room temperature. All pyrite particles were sieved through 200 mesh (74 μm)^35,49^. Afterward, the pyrite powder was mixed with a specific quantity of Ti(C_4_H_9_O)_4_ dissolved in ethanol to synthesize TiO_2_/NP. The resulting mixture was stirred with a magnetic stirrer for 2 h before being dried at 80 °C. Finally, The dried as-prepared was then heated to 550 °C for 2 h and cooled to room^35^.

### Characterization

XRD analyses were carried out to crystalline phase determination of synthesized catalysts. The surface morphology and chemical composition of pyrite, and the synthesized nanocomposite were determined by Scanning Electron Microscopy (SEM) equipped with energy-dispersive X-ray spectroscopy chemical analysis (EDS). Fourier transforms infrared spectroscopy (FTIR) was conducted in the range of 4,000 to 500 cm^-1^ to determine functional groups on the surface of the catalyst. The diffuse reflectance spectroscopy (DRS) technique was used to analyze the optical properties of the samples. The details of XRD, FTIR, and SEM–EDX techniques were described in previous study^35^.

### Tetracycline degradation using photocatalytic approach

The elimination of TC was investigated using the photocatalytic approach in a 500 ml rectangular, black-colored, plexiglass reactor containing a 100 ml sample. Four LED lamps with a maximum wavelength of 450 nm and 15mW/cm^2^ intensity were installed in the central upper part of the reactor. All experiments took place in a batch system. The photocatalytic potential of the nanocomposite was assessed by introducing a particular quantity of the catalyst into the solution. Later adjusting the pH value of the solution with hydrochloric acid and sodium hydroxide (0.01 M), the visible lamps were turned on and the samples were mixed at 200 rpm with a magnetic stirrer for the required time. Afterward, 1 ml of sample was extracted at predetermined intervals. The remaining level of the residual pollutant was measured by utilizing high-performance liquid chromatography (HPLC) with an Agilent 1200 Infinity Series instrument from the United States. The effect of varying process parameters including pH (3, 7, and 9), TiO_2_/pyrite dosage (0.1, 0.5, 1, 2, and 3 g/L), initial tetracycline concentration (10, 20, 30, 40 and 50 mg/L), and the intensity of visible light (15, 30, and 60 mW/cm^2^) on TC removal were examined. Total organic carbon (TOC) analysis was conducted in order to determine the mineralization value during pollutant degradation by photocatalytic approach. Furthermore, tert-butanol (0.5 mmol/L), potassium iodide (0.5 mmol/L), and Cr (VI) (0.5 mmol/L) were employed to examine the impact of the reactive species generated throughout the photocatalytic reaction.

## Results and discussion

### Catalyst characterization

**Figure 1:**
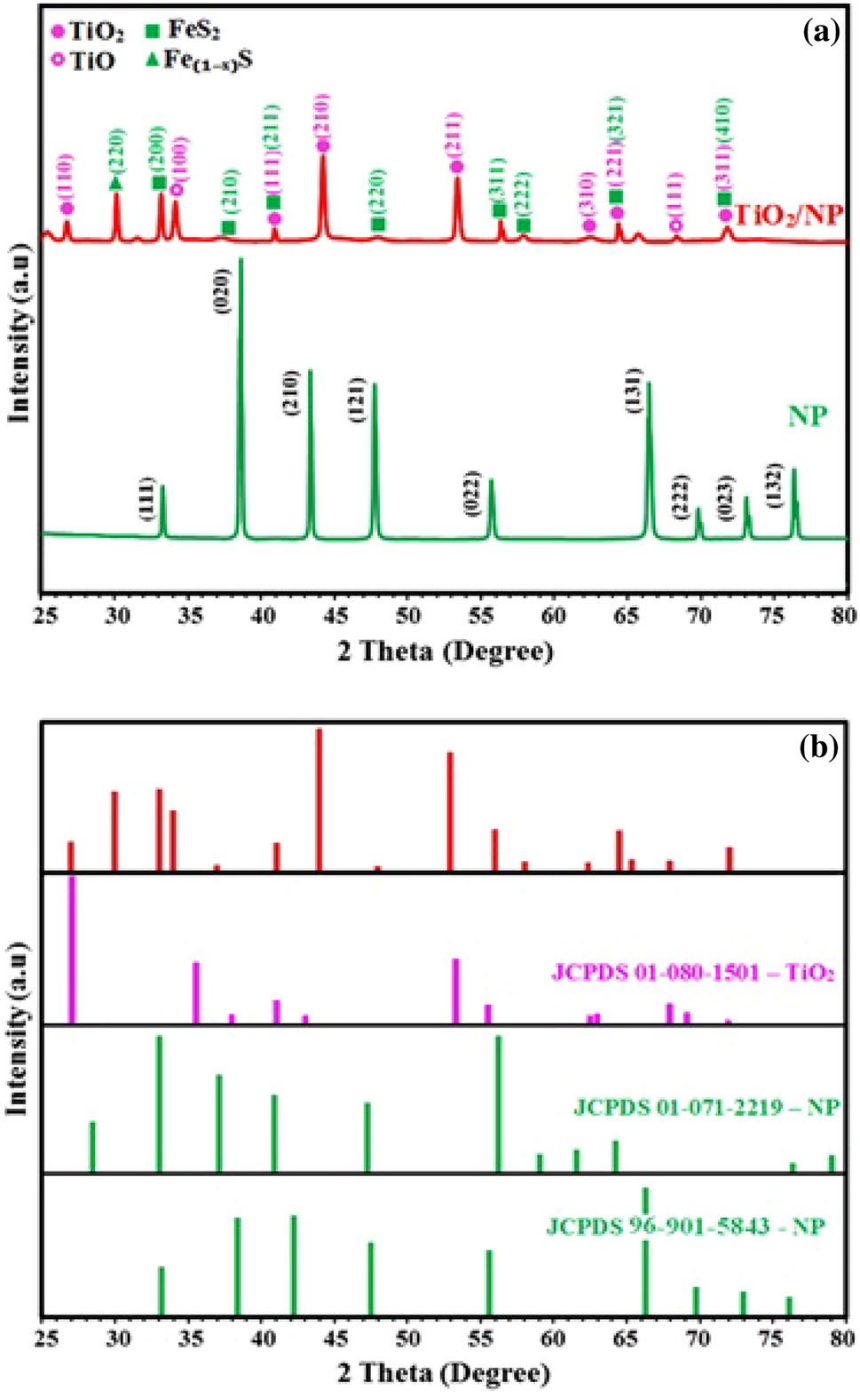
(**a**) and (**b**) XRD patterns of NP and TiO_2_/NP.

The details of XRD, FTIR, and SEM–EDX results were described in previous study^35^. XRD patterns of natural pyrite (NP) and TiO_2_/NP are shown in Fig. 1a and b. The findings demonstrated that following calcination during the TiO_2_/NP synthesis process, the pyrite's XRD pattern had slightly altered. The intensity of the peak at 2θ = 73.13° decreased, while the peak at 2θ = 76.37° faded. The results also showed that pyrrhotite (Fe_(1 − x)_S) was allocated to the peak at 2θ = 30° (220)^50^. The emergence of pyrrhotite could be attributed to the influence of the calcination procedure on the configuration of FeS_2_^51^. Furthermore, XRD analysis has verified significantly intensified pyrite diffraction peaks within the TiO_2_/NP sample. This observation suggests that there is a notable concentration of pyrite present in the TiO_2_/NP^35,52^.

**Figure 2:**
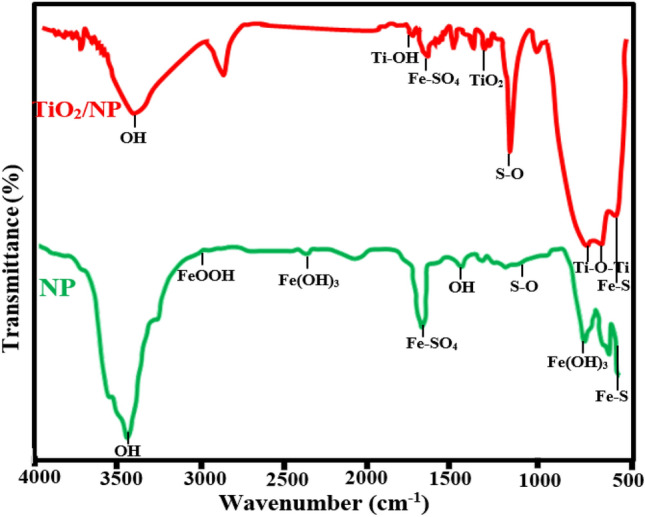
FTIR spectrum of NP and TiO_2_/NP.

Figure 2 depicts the FTIR distinctive peaks of TiO_2_/NP. The details of FTIR spectra of pyrite and synthesized catalyst were presented in our previous study^35,53^. FTIR spectra of TiO_2_/NP indicate that the two peaks around 2360 and 2925 cm^−1^ observed in pyrite are attributed to iron (III) hydroxide and goethite, respectively; these peaks were diminished subsequent to the decoration of TiO_2_ with pyrite. The findings suggest a decrease in the Fe_2_O_3_ coating on the NP^35^. The emergence of the peak observed at 1630 cm^-1^ is ascribed to the Ti–OH functional group, which implies the existence of TiO_2_ on the pyrite's surface^54^.

**Figure 3:**
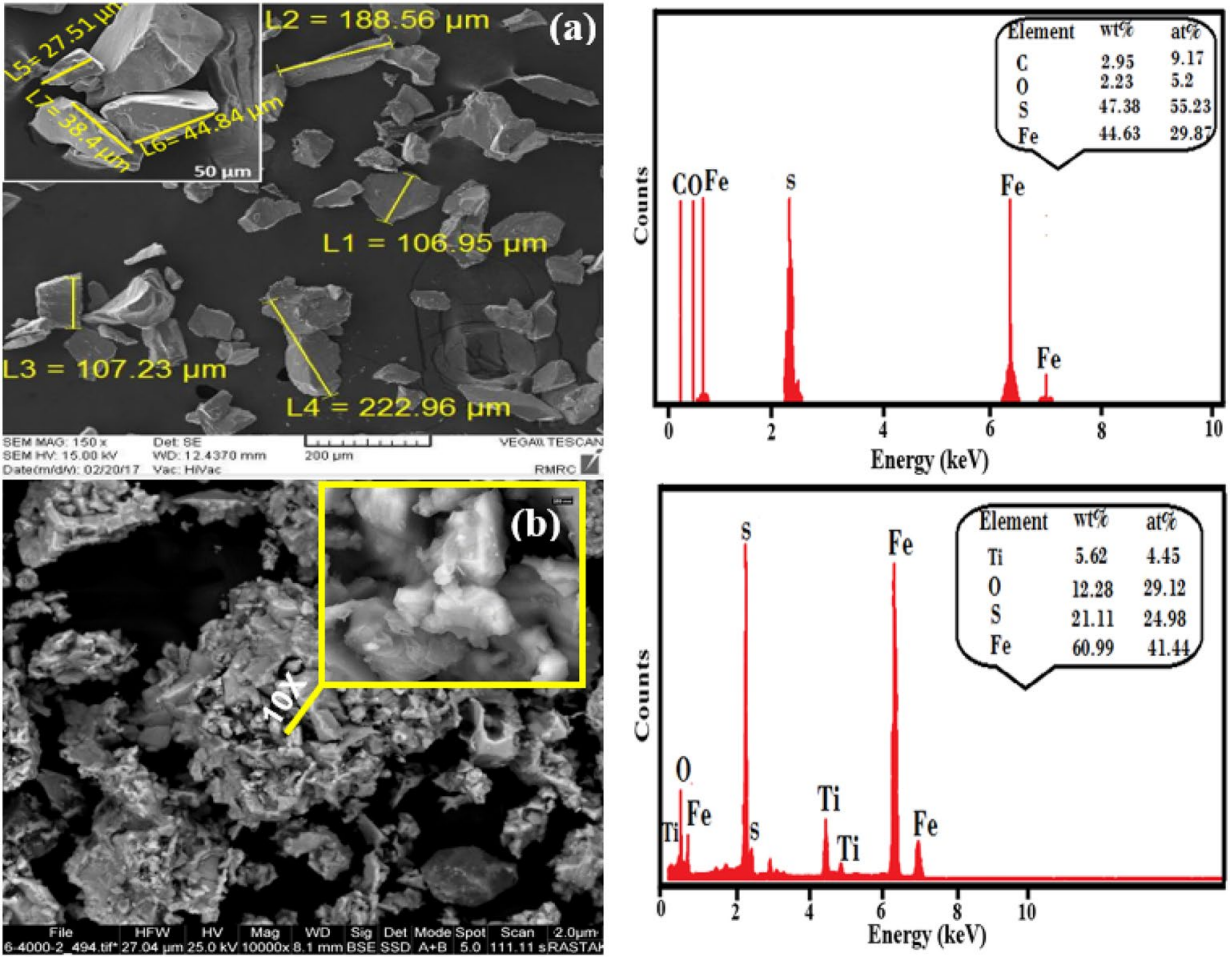
The image of SEM–EDS (**a**) NP and (**b**) TiO_2_/NP.

The morphological characteristics of pyrite and TiO_2_/NP are depicted in Fig. 3a,b. As illustrated in Fig. 3a, the natural massive structure and irregular exterior surface of the pyrite enhance its surface area. This exposes more of the iron present on the catalyst surface, facilitating a high quantity formation of ROS^55^. EDS results (Fig. 3a) show that sulphur (S) and iron (Fe) are the predominant elements, while oxygen (O) and carbon (C) are present. The attendance of C and O could be assigned to the adsorption of organic substances or iron oxide during grinding and milling. SEM analysis (Fig. 3b) reveals that pyrite particles are composed of TiO_2_ and exhibit agglomerated and irregular features. The successful immobilization of TiO_2_ onto the pyrite surface leads to a lustrous color and reduced sharpness of pyrite edges. The synthesis of TiO_2_/NP was confirmed by the detection of Ti, O, S, and Fe in the catalyst. Additionally, the weight percentage of oxygen increased after impregnation, indicating the presence of oxygen within the TiO_2_ crystal structure^56^.

**Figure 4:**
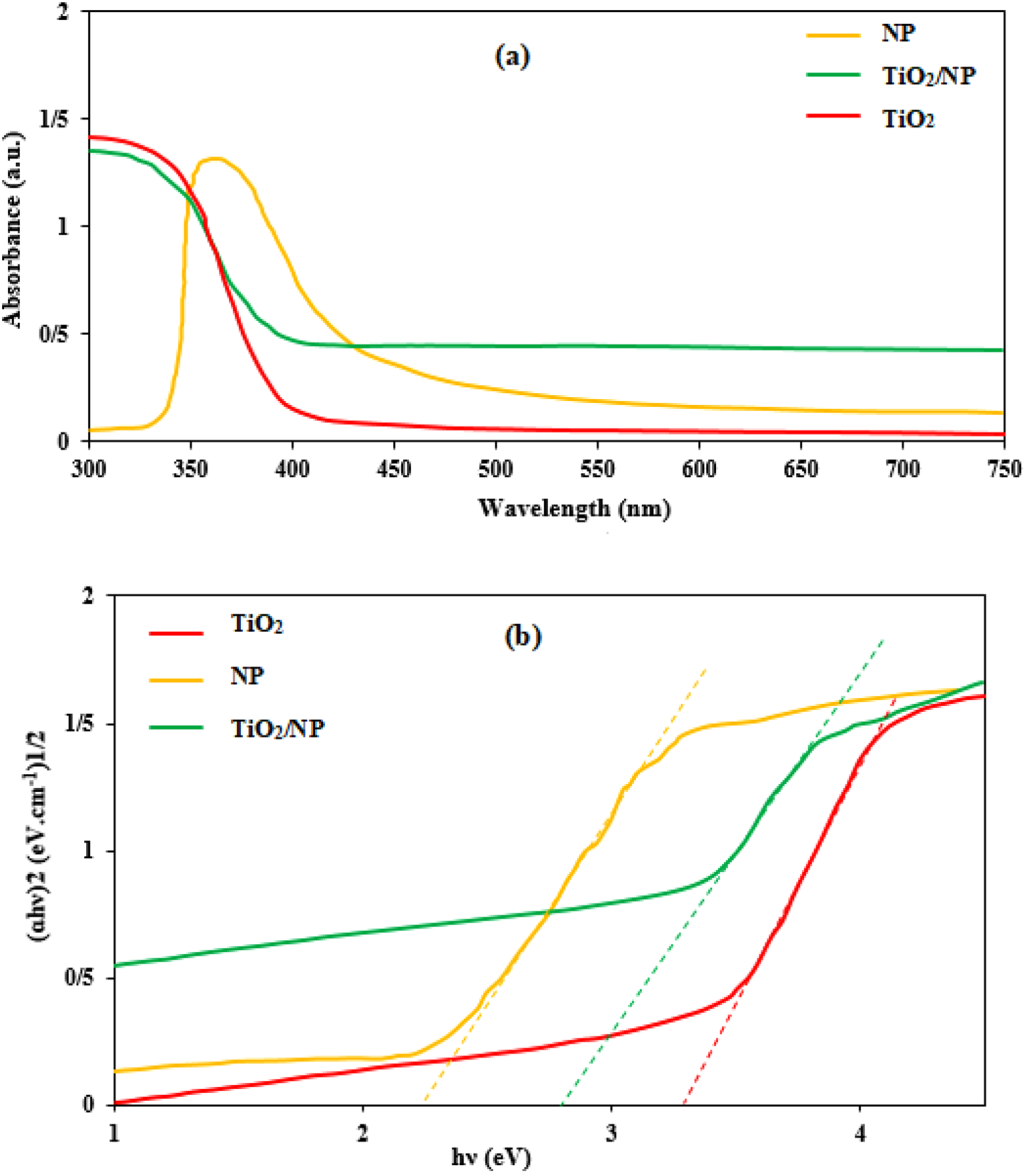
The DRS results of TiO_2_, NP and TiO_2_/NP.

The optical properties of NP and TiO_2_/NP were analyzed using the DRS technique, as shown in Fig. 4a,b. The results revealed that the absorbance spectra of NP and TiO_2_/NP were observed at 810 nm and 490 nm, respectively. These values correspond to the band gaps of NP and TiO_2_/NP, which are determined to be 2.23 eV and 2.84 eV, respectively. The findings confirm that NP can reduce the band gap of TiO_2_, resulting in an increased generation of ROS when exposed to visible light irradiation.

### Impact of varying reaction conditions on photocatalytic process

#### Influence of pH

The photocatalytic process is significantly influenced by pH due to several factors, including alterations in the catalyst surface charge, the degree of ionization, and the formation of diverse pollutant species at varying pH levels^1,16^. Therefore, the impact of the initial pH on the photocatalytic elimination of TC was evaluated across three distinct pH levels, including acidic, neutral, and basic.

**Figure 5:**
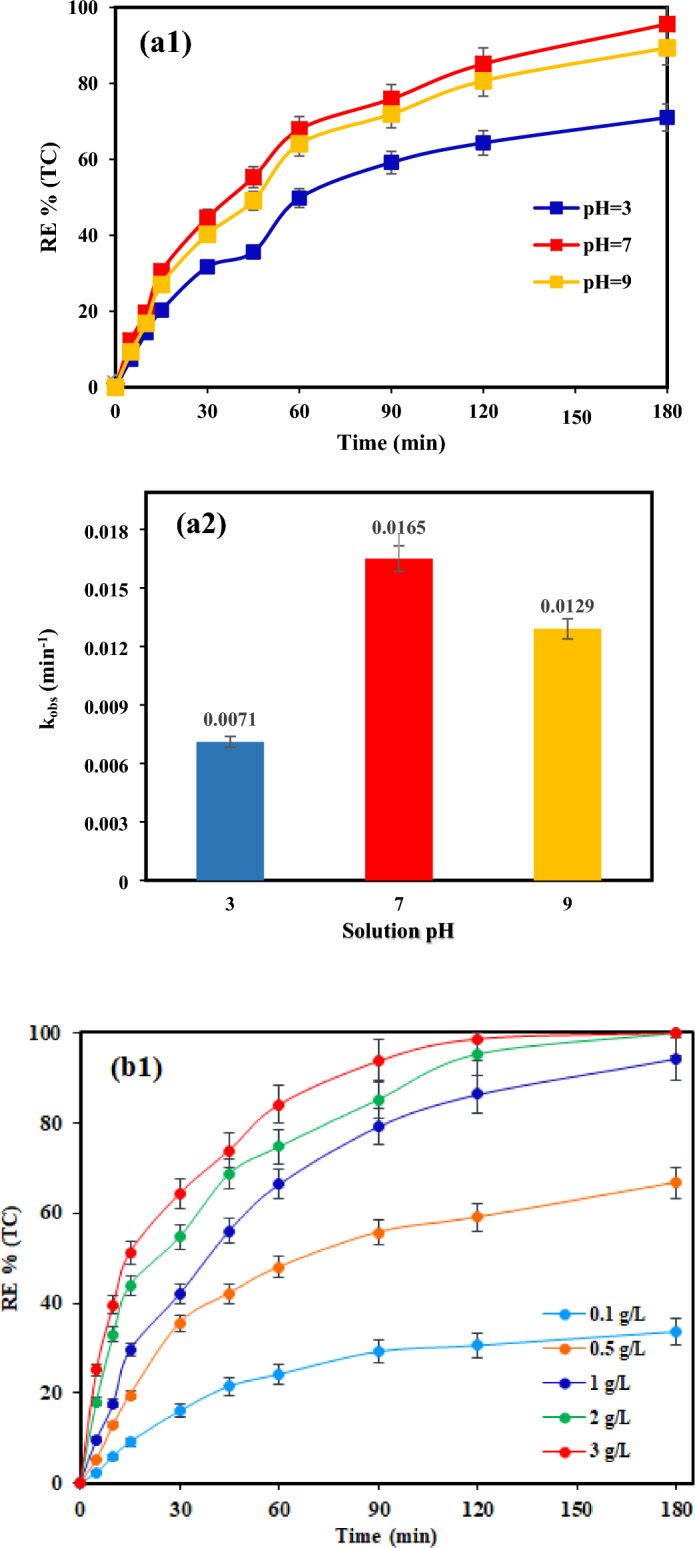

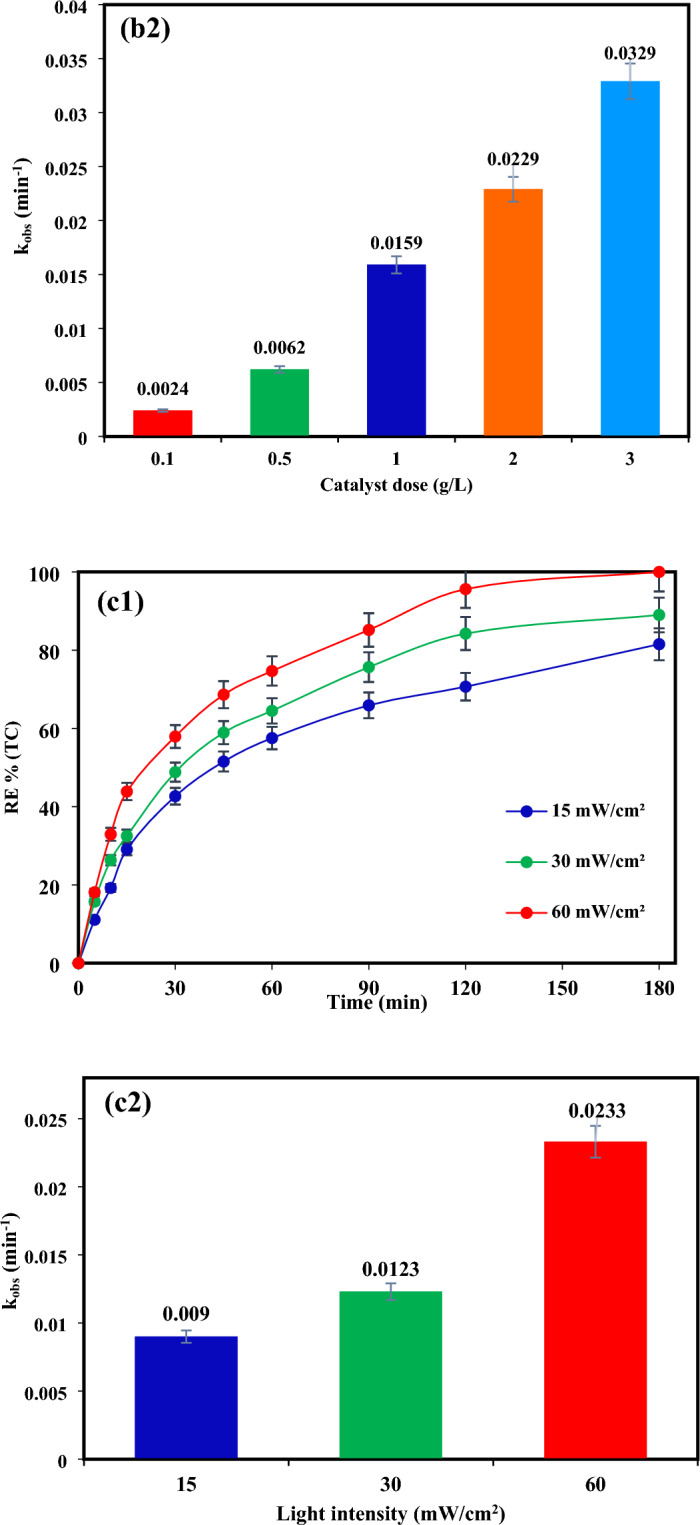

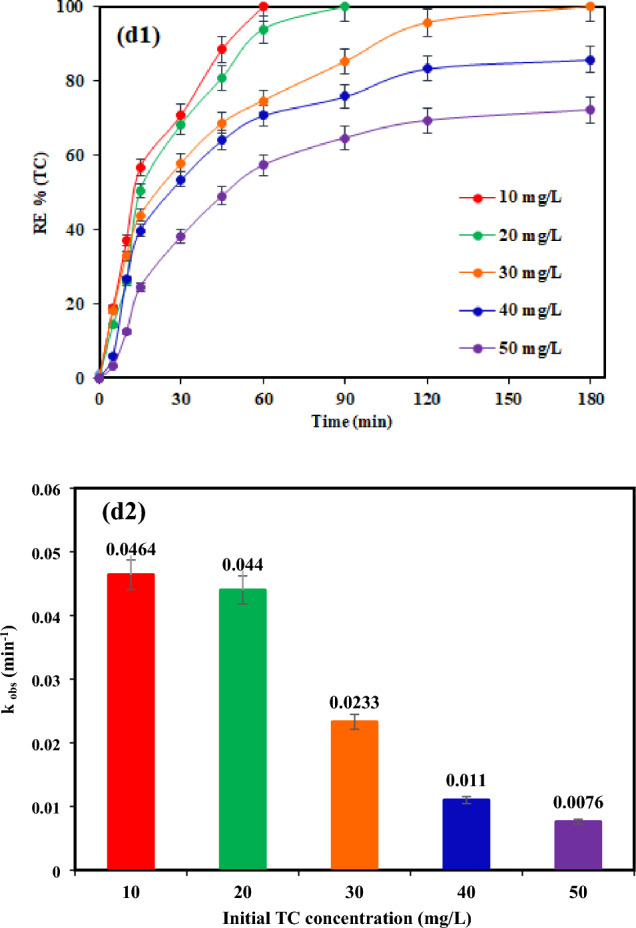
Effects of operational parameters and variation profiles of pseudo-first-order rate constants of TC degradation: (**a**) initial pH; (**b**) catalyst dose; (**c**) light intensity; (**d**) TC concentration (Reaction conditions: (**a**): catalyst dose = 1 g/L, [TC] = 30 mg/L and light intensity = 60 mW/cm^2^; (**b**): ([TC] = 30 mg/L, pH = 7 and light intensity = 60 mW/cm^2^; (**c**): TC] = 30 mg/L, pH = 7 and catalyst dose = 2 g/L; (**d**): pH = 7 and catalyst dose = 2 g/L and light intensity = 60 mW/cm^2^).

Figure 5a1, a2 demonstrate that the most significant removal of TC was observed at a neutral pH, with a corresponding k_obs_ = 0.0165 min^−1^. As mentioned, as well as alterations in the surface charge of catalyst, different forms of TC are generated as the pH values change^6,57^. The photocatalyst demonstrates low TC adsorption at different pH levels, resulting in only 26.7%, 63.5%, and 48.1% of TC being adsorbed by TiO_2_/NP at pH 3, 7, and 9 respectively. The adsorption process depends on the nature of the adsorbent and adsorbate. TC has three pK_a_ values: 3.3, 7.7, and 9.7, which can affect its hydrolysis and precipitation processes. At 3.3 < pH < 7.7, the molecular structure of TC is TCH_3_^+^; at 7.7 < pH < 9.7, it is TCH^0^_2_ and TCH¯; and at pH > 9.7, it takes the TCH^2^¯ form. Since the point of zero charge (pH_pzc_) of TiO_2_/pyrite is 6.95 under acidic conditions (pH < pH_pzc_), the composite had a positive surface charge, while, at acidic pH, TC existed in the positive form (TCH^3+^)^57^. Consequently, the presence of a solid repulsive force between the positively charged ions and the charged surface of the TiO_2_/NP hindered the adsorption process of ions on the surface of the catalyst. This resulted in a decrease in the TC removal under acidic pH conditions^22,58^. The decline in photocatalytic efficacy at basic pH may be ascribed to the electrostatic repulsion between the negative surface charge of the catalyst (pH > pHpzc) and the negative ions of TC that are generated at higher pH levels^59–61^. At a pH of 7, the concentrations of positive and negative charges are nearly balanced. However, due to the minimal presence of negative charge on the catalyst surface at pH 7, and the predominance of TC molecules in the TCH^3+^ form, maximum absorption has occurred. Moreover, in the photocatalytic process, surface hydroxyle radicals, in addition to bulk hydroxyle radicals, are generated, which decompose the absorbed molecules.

Although alkaline pH conditions generate a greater quantity of the ^·^OH, that can enhance the removal process as a reactive oxidative species (ROS), the photocatalytic efficiency is diminished as a consequence of the ferrous and ferric precipitation and the generation of Fe(OH)_2_^4+^. This is attributed to its lower catalytic activity in comparison to Fe^2+^^62^. Conversely, the deposition and existence of these hydroxides at the catalyst surface hinder the photocatalytic elimination of TC^62^. Based on the findings, the neutral pH was elected as the optimum pH for further investigations.

#### Influence of TiO_2_/NP concentration

The findings from investigating the impact of TiO2/NP concentration on TC elimination (as depicted in Fig. 5b1) indicate that as the photocatalyst concentration raised from 0.1 to 2 g/L, the degradation efficacy of TC improved significantly, rising from 30.67% to 100% over a 180-min timeframe. According to the data presented in Fig. 5b2, an increase in catalyst dosage from 0.1 to 3 g/L resulted in a rise in the reaction rate constant (k_obs_) from 0.0024 to 0.0329 min^−1^.

The rise in active sites on the photocatalyst surface is believed to be the cause of the observed phenomenon^63^. However, adding a photocatalyst dosage more than 2 g/L had no significant effect on degradation efficiency. Therefore, according to economic considerations and appropriate degradation efficacy, the quantity of 2 g/L of TiO_2_/NP was determined as the optimal value for further photocatalytic experiments.

On the one hand, when the concentration of the photocatalyst is increased, it leads to an expansion of its surface area and a rise in the quantity of active sites. Consequently, a higher quantity of pollutant molecules and light photons can attach to the photocatalyst surface, increasing electron excitation from the valence band to the conduction band^1^. Furthermore, the amount of ^•^OH and superoxide radicals that enhances the photocatalytic reaction could undergo an increase^5,64^.

On the other hand, at higher photocatalyst dosage, the augmented opacity of the solution impedes the infiltration of light on the surface of the photocatalyst^38^. Also, an overabundance of light scattering resulted in a decline in the ^•^OH generation, ultimately resulting in a decrease in the photocatalytic efficacy^64^. Furthermore, catalyst loading on the surface beyond the optimum led to agglomeration of catalyst^4,61^. Thereby, the active sites of the catalyst surface become inaccessible for pollutants and photons absorption.

Ahmadi et al. (2017) discovered that the dosage of the catalyst can have both positive and negative impacts on phenol degradation in the photocatalytic approach. According to the findings of their study, the elimination efficiency of phenol was enhanced after 100 min when the concentration of MWCNT/TiO_2_ nanocomposite was raised from 0.1 to 2 g/L. However, no rise in removal efficiency was observed when the concentration was set at 4 g/L, at the same reaction time. The researchers suggested that increasing the concentration of the catalyst could reduce active site access to light photons, resulting in a loss in photocatalyst overall efficiency^61^.

#### Influence of visible light intensity

The effectiveness of photocatalytic elimination of pollutants is significantly influenced by the intensity of light. At optimal catalyst concentration, the influence of various visible light intensities (15, 30, and 60 mW/cm^2^) for TC removal was examined. As shown in Fig. 5 (c1,c2), by raising the irradiation intensity from 15–60 mw/cm^2^, the TC removal efficiency has improved from 82 (with the k_obs_ = 0.009 min^−1^) to 100% (with the k_obs_ = 0.0233 min^−1^) after 180 min. According to the results, TC degradation is positively impacted by light intensity. Because light intensity is required for the photochemical reaction to begin, electron–hole pairs (e^-^/h^+^) production predominates and electron–hole recombination is insignificant at higher light intensities^64,65^. Furthermore, the number of light photons at higher intensity increased, which can also increase the photocatalytic decomposition of TC^5,66^. Nevertheless, the lower decomposition efficiency observed at lower light intensity can be assigned to the competition between the separation of e^-^/h^+^ pairs and their recombination. This competition results in reduced ROS formation and as a consequence less degradation of contaminants^67^.

#### Influence of TC concentration

Experiments were conducted under ideal conditions with varied TC concentrations ranging from 10 to 50 mg/L to evaluate the influence of the initial concentration of TC on the photocatalytic effectiveness of TiO_2_/NP. The efficacy of TC removal diminished as the initial concentration increased, as depicted in Fig. 5(d1). This decrease was further supported by the decline in k_obs_ values (Fig. 5d2) from 0.0464 to 0.0076 min^-1^ when the concentration of TC was elevated from 10 to 50 mg/L.

The reduction of active sites on the catalyst surface may be responsible for this phenomenon. When the catalyst dosage is kept constant, the quantity of active sites on the catalyst surface remains unchanged^38^.

As the amount of pollutants increases, so does the number of pollutant molecules that may be present on the active sites of the catalyst surface. Consequently, the availability of active sites for this higher number of pollutant molecules will be less^6,64^. In addition, as the concentration of pollutant increase, more TC molecules or the formed intermediates could be adsorbed on the photocatalyst surface. Therefore, more active oxidant species are required to remove this high pollutant concentration, and their insufficiency escalates proportionally with the augmentation of TC amount^4,61^. Conversely, Wang et al. (2020) suggested that an overabundance of pollutants adsorbed onto the photocatalyst surface can impede the transmission of optical photons to the photocatalysts surface. Thereby, the efficacy of the photocatalytic process is impeded by the deficiency of active oxidants including hydroxyl radicals and superoxide anions (O^·−^_2_)^59^.

### Performance of various processes on TC removal

**Figure 6:**
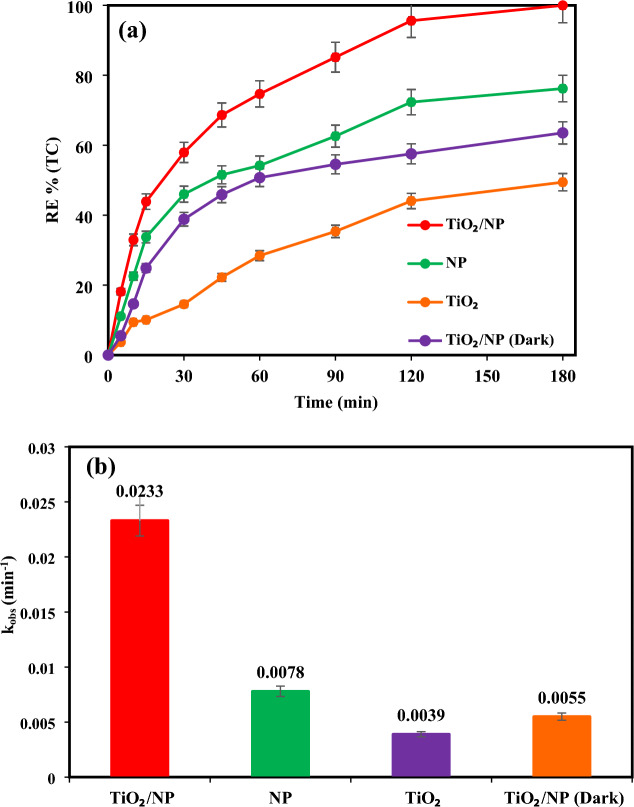
(**a**) TC Removal efficiency under different systems at optimal conditions ([TC] = 30 mg/L, pH = 7, catalyst doe = 2 g/L and light intensity = 60 mW/cm^2^), (**b**) variation profiles of pseudo-first-order rate constants of TC degradation.

**Figure 7:**
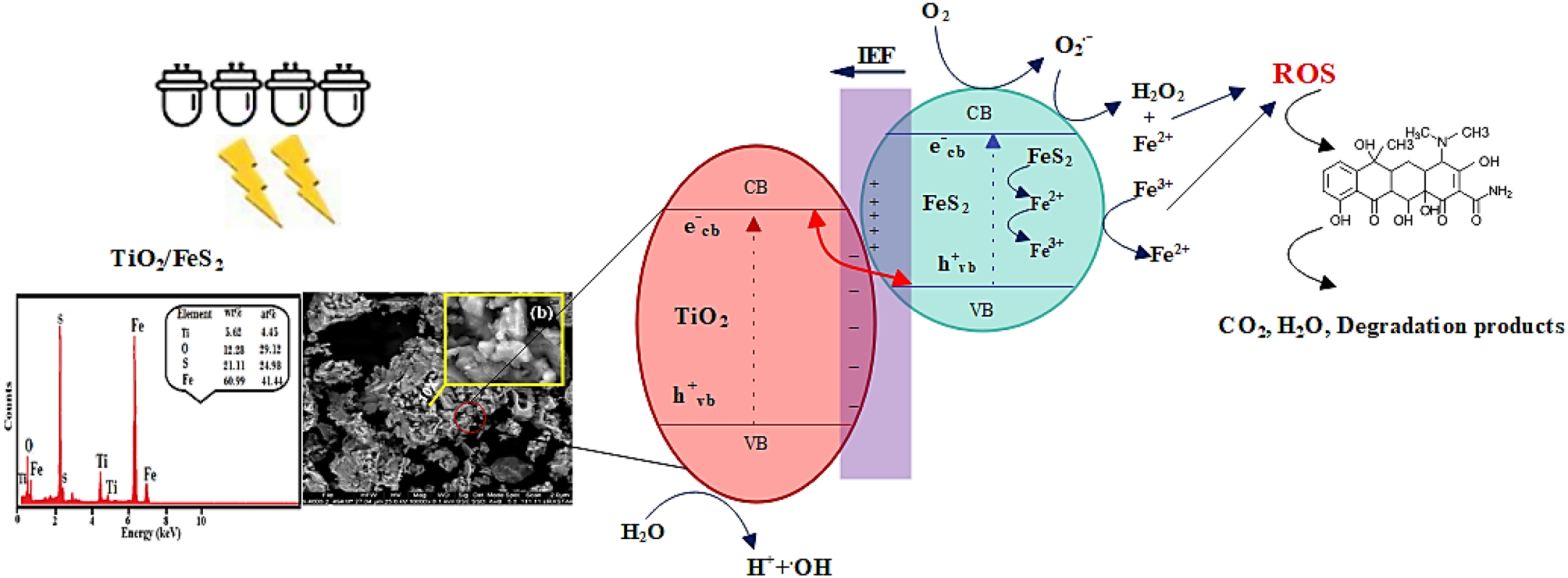
The schematic illustration of photocatalytic degradation of TC using TiO_2_/NP.

To examine the efficacy of TiO_2_ or pyrite in TC removal, photocatalytic experiments were conducted in the presence of TiO_2_ and pyrite alone at optimal conditions (pH 7, catalyst dosage of 2 g/L, TC concentration of 30 mg/L, and light intensity 60 mW/cm^2^), findings were subsequently compared with those of the TiO_2_/NP system. Moreover, the impact of TiO_2_/pyrite on TC elimination was explored in the absence of light. As demonstrated in Fig. 6a, the binding of TC on the TiO_2_/NP surface could explain the elimination of TC in the absence of visible light. After 180 min of reaction time in a TiO_2_-based system, TC elimination effectiveness was 63.54%. Meanwhile, a single pyrite system exhibited higher removal efficiency of 77.87% for TC throughout the same reaction time. On the contrary, coating TiO_2_ on the surface of pyrite (Fig. 7) resulted in higher photocatalytic efficiency than in the presence of TiO_2_ and pyrite alone. Also, as it can be seen, the efficiency of pyrite in photocatalytic removal of TC is greater than that of TiO_2_. The dissimilarity between the band gaps of TiO_2_ (3.2 eV)^59^ and pyrite (0.95 eV)^35,68^, which causes a difference in the absorption of light photons and more oxidant species generation in the photocatalytic process in the attendance of pyrite, may be the cause of the differences in removal efficiencies between these two substances^35^. Indeed, the higher Fermi level of pyrite compared to TiO_2_ causes electrons to transfer from pyrite to TiO_2_ leading to an internal electric field between pyrite and TiO_2_. Under the illumination of light, in accordance with the S-scheme photocatalyst approach, pyrite functions as the reduction photocatalyst (RP), while TiO_2_ acts as the oxidation photocatalyst (OP). As a result, the photogenerated electrons gather in the conduction band (CB) of pyrite, while the photogenerated holes accumulate in the valence band (VB) of TiO_2_. This process reduces the recombination of electron–hole pairs with a high potential, thereby enhancing the photocatalytic activity of the TiO_2_/NP nanocomposite. Additionally, the low potential photogenerated holes in the valence band of pyrite are recombined by the photogenerated electrons in the conduction band of TiO_2_, facilitated by the internal electric field. Consequently, the photogenerated electrons in the conduction band of pyrite and the holes in the valence band of TiO_2_ are effectively utilized for TC reduction, resulting in a higher photocatalytic efficiency of TiO_2_/NP for TC degradation^69–71^.

In addition, the increased efficacy of pyrite in TC removal compared to TiO_2_ can be described by the formation of ferrous ions^57,72^. According to the following equation (Eq. (1)), first, the Fe in the solution is in a state of Fe(OH)^2+^. Under the irradiation of light, it is converted into the forms of Fe^2+^ and ^•^OH, thus providing one of the essential oxidant species for photocatalytic oxidation.

1 $${{Fe(OH)}}^{2+}+\text{ h}\vartheta \, \to \, {{Fe}}^{2+}+{\cdot }{\text{OH}}$$

As observed in Fig. 6b, the reaction rate constant of TC decomposition was significantly rose in TiO_2_/NP system under visible light irradiation (0.0233 min^−1^).

As mentioned above, since pyrite has a relatively small band gap, the immobilization of TiO_2_ on pyrite causes an improvement in the photocatalytic efficacy of TiO_2_ during visible light exposure. In the conduction and valence bands of pyrite, photoelectrons (e^−^) and positive holes (h^+^) are produced under visible light, respectively^52,73^. Furthermore, the H^+^ and ^•^OH radicals can be generated as a result of H_2_O and positive holes (h^+^) reaction. (Eqs. (2)–(8))^35^. Therefore, the existence of pyrite and TiO_2_ causes the formation of additional oxidative species, which improves the effectiveness of TC removal^74^. The TiO_2_/NP photocatalytic efficiency was compared with previously researched photocatalysts, as demonstrated in Table 3.

**Table 3:** Comparison of TC photocatalytic removal using the different photocatalyst.

No	Catalyst	Light source	Experimental conditions	Efficiency (%)	Ref
1	C_3_N_4_-TE@TiO_2_/UiO-66	Visible light	[C_0_]:20 mg/L, [catalyst]: 0.4 g/L, [pH]:4, [Time]:40 min	96	^26^
2	Fe-MOF/CM	Visible light	[C_0_]:10 ppm, [catalyst]:0.375 g/L, [Time]:60 min	100	^75^
3	FeNi_3_@SiO_2_@TiO_2_	UV-light	[C_0_]:10 mg/L, [catalyst]: 0.005 g/L,[pH]:9, [Time]:200 min	100	^1^
4	Calcite/TiO_2_	UV-light	[C_0_]:50 mg/L, [catalyst]:1.5 g/L, [pH]:7, [Time]:300 min	> 90	^16^
5	TiO2/Mo_9_O_28_-BMPP	Visible light	[C_0_]:25 mg/L, [catalyst]: 0.15 g, [pH]:7, [Time]:115 min	97	^59^
6	MWCNT/TiO_2_	UVC-light	[C_0_]:10 mg/L, [catalyst]: 0.2 g/L, [pH]:5, [Time]:300 min	100	^61^
7	TiO_2_	Visible light	[C_0_]:10 mg/L, [catalyst]: 0.2 g/L , [Time]: 120 min	56.7	^23^
8	AgI/UiO-66(NH_2_)	Visible light	[C_0_]:10 mg/L, [catalyst]: 0.3 g/L, [pH]:4.3, [Time]:40 min	80.7	^6^
9	In-doped Mn_2_O_3_	Visible light	[C_0_]:50 mg/L, [catalyst]:10 mg/L, [pH]:7, [Time]:180 min	93	^63^
10	MoS_2_/ZnSnO_3_	Visible light	[C_0_]:30 mg/L, [catalyst]:0.25 g/L, [Time]:60 min	80.2	^18^
11	BiVO_4_/FeVO_4_@rGO	Visible light	[C_0_]:30 mg/L, [catalyst]:25 mg, [pH]:2, [Time]:100 min	91.5	^76^
12	Ag_3_PO_4_/AgBr/g-C_3_N_4_	Visible light	[C_0_]:10 mg/L, [catalyst]:0.5 g/L, [pH]:7, [Time]:10 min	90	^77^
			[C_0_]:40 mg/L, [catalyst]:0.5 g/L, [pH]:7, [Time]:25 min	80.2	
13	AgBr-TiO_2_-Pal	Visible light	[C_0_]:10 mg/L, [catalyst]:0.5 g/L, [pH]:9, [Time]:90 min	90	^22^
14	CDs-ZnSnO_3_	Visible light	[C_0_]:10 mg/L, [catalyst]:0.25 g/L, [Time]:60 min	81.7	^78^
15	TiO_2_/NP	Visible light	[C_0_]:30 mg/L, [catalyst]:2 g/L, [pH]:7, [Time]:180 min	100	This study

2 $${{\text{FeS}}}_{2}+\mathrm{h\vartheta }\to {{\text{FeS}}}_{2} ({{\text{e}}}^{-}+ {{\text{h}}}^{+})$$

3 $${{\text{TiO}}}_{2}/{{\text{FeS}}}_{2} ({{\text{e}}}^{-}+ {{\text{h}}}^{+}) \, \to {{\text{FeS}}}_{2} \left({{\text{h}}}^{+}\right) +{{\text{TiO}}}_{2}({{\text{e}}}^{-})$$

4 $${{\text{h}}}_{{\text{VB}}}^{+}+{{\text{H}}}_{2}{\text{O}}\to {{\text{H}}}^{+}{+}{\cdot }{\text{OH}}$$

5 $${{\text{O}}}_{2}+ {{\text{TiO}}}_{2}({{\text{e}}}^{-})\to {{\text{O}}}_{2}^{\cdot -}$$

6 $${{\text{O}}}_{2}^{\cdot -}+{{\text{FeS}}}_{2} \left({{\text{h}}}^{+}\right) \to {{\text{HO}}}_{2}^{\cdot }$$

7 $${{\text{TiO}}}_{2}\left({{\text{e}}}^{-}\right)+ {{\text{HO}}}_{2}^{\cdot }\to {\text{H}}_{2}{{\text{O}}}_{2}$$

8 $${{\text{TiO}}}_{2}\left({{\text{e}}}^{-}\right)+ {\text{H}}_{2}{{\text{O}}}_{2}\to {\text{OH}}^{-}\text{+}\cdot {\text{OH}}$$

9 $${\text{e}}_{\text{CB}}^{-}\text{ } + {\text{ O}}_{2}\to {\text{O}}_{2}^{\cdot \text{ } - }$$

10 $${\text{2O}}_{2}^{\cdot \text{ } - }\text{ + }{\text{2H}}^{+}\to {\text{ O}}_{2}+ \text{ } {\text{H}}_{2}{{\text{O}}}_{2}$$

11 $${\text{H}}_{2}{{\text{O}}}_{2}\text{+}{\text{Fe}}^{2+} \, \to \, {\text{Fe}}^{3+}\text{+}{\text{OH}}^{-}\text{+}\cdot {\text{OH}}$$

12 $${\text{H}}_{2}{{\text{O}}}_{2}\text{+}{\text{Fe}}^{3+} \, \to \, {\text{Fe}}^{2+}\text{+}{{\text{H}}}^{+}\text{+}{{\text{HO}}}_{2}^{\cdot }$$

13 $${{\text{HO}}}_{2}^{\cdot } \to {{\text{O}}}_{2}^{\cdot -}+ {{\text{H}}}^{+}$$

14 $${{\text{O}}}_{2}^{\cdot -}+{\text{H}}_{2}{{\text{O}}}_{2}\to \cdot {\text{OH}}+{\text{O}}_{2}$$

15 $${\text{H}}_{2}{{\text{O}}}_{2} +{e}_{CB}^{-} \to {\text{OH}}^{-}\text{+}\cdot {\text{OH}}$$

16 $${\text{H}}_{2}{{\text{O}}}_{2} +h\vartheta \to {2}\cdot {\text{OH}}$$

### Reactive species identification

**Figure 8:**
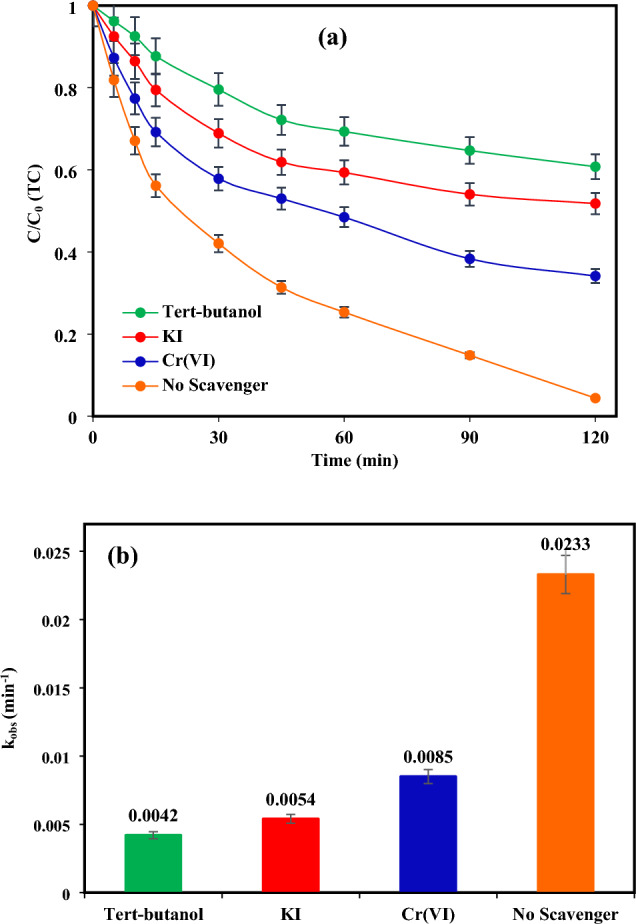
(**a**) The influence of various scavengers and (**b**) the reaction rate constants associated with those scavengers under ideal circumstances ([TC] = 30 mg/L, pH = 7, catalyst doe = 2 g/L and light intensity = 60 mW/cm^2^).

The effectiveness of the photocatalytic system is significantly influenced by the presence of oxidative species. To determine the most significant reactive oxidation species, quenching agents such as TBA, KI, and Cr (VI) with a concentration of 0.5 mM were utilized as scavengers of ^•^OH, h^+^, and e^−^, respectively. As presented in Fig. 8a, in the absence of scavengers, 95.6% of the target compound (TC) was eliminated within 120 min of photocatalytic treatment. Nevertheless, the removal efficacy of TC declined in the attendance of all radical scavengers. The presence of TBA causes a remarkable reduction in the removal efficacy of TC, with a reaction rate constant (k_obs_) (Fig. 8b) of 0.0042 min^−1^. This suggests that hydroxyl radicals (^·^OH) are the primary oxidative species involved in the photocatalytic decomposition of TC, compared to other active species.

This data implies that the attendance of TBA reduced the photocatalytic system's efficacy, underlining that ^•^OH radicals are major involved ROS in the elimination of TC^57,79^. The observed effect may be due to TBA's increased aqueous solubility and lower absorption rate by the catalyst, allowing it to swiftly scavenge free radicals^80^. Hence, ^•^OH > h^+^  > e^-^ are the influential oxidation species participating in the photocatalytic elimination of TC by TiO_2_/NP composite.

### Effects of inorganic ions on TC removal

**Figure 9:**
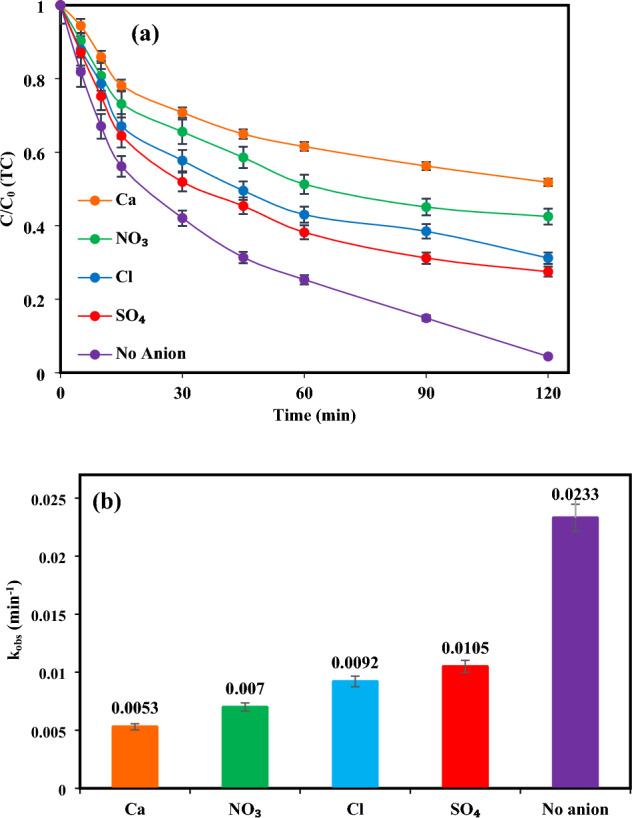
(**a**) TC Removal rate and and (**b**) the corresponding reaction rate constants at optimum conditions ([TC] = 30 mg/L, pH = 7, catalyst doe = 2 g/L and light intensity = 60 mW/cm^2^).

Since the practical application of photocatalytic oxidation may be influenced by inorganic ions, which widely exist in aqueous matrices, indeed, the photocatalytic efficiency of a TiO_2_/NP system for TC removal was evaluated under optimal conditions in the existence of Ca^2+^, NO_3_^-^, SO_4_^2−^, and Cl^-^ ions at a concentration of 20 mM. According to the results (Fig. 9 (a,b)), it can be observed that the attendance of different ions has resulted in a decrease in the removal of TC in comparison to water without ions. Furthermore, the restrictive impact of these ions has been observed to follow the order of Ca^2+^ > NO_3_^-^ > Cl^-^ > SO_4_^2−^. This phenomenon is caused by the assimilation of light photons by these ions, which impede the photolysis of additional water molecules and the establishment of hydroxyl radicals. Consequently, the quantity of ^•^OH radicals available for the oxidation of TC is reduced. This is because the scavenging agents (such as TBA, KI, and Cr(VI)) react with the ^·^OH radicals, resulting in a decline in their concentration and subsequent reduction in the overall efficacy of the photocatalytic system^81^. Furthermore, the existence of inorganic ions may lead to the occupation of active sites present on the TiO_2_/NP surface, which can result in a decline in the accessibility of TC molecules due to competition with these ions. This causes a decline in the photocatalytic efficacy of the system since fewer active sites are obtainable for the oxidation of TC molecules^79^. Conversely, the reaction of ions with the main reactive species (^·^OH, h^+^), which leads to the generation of radicals with lower oxidative potential, can be another reason for the reduction in the oxidation process^79,82^.

As shown, the highest hindering effect was related to calcium ions, possibly due to the consumption of hydroxyl ions and bonding with ferrous ions^83^. The addition of nitrate ions significantly reduces TC elimination because these ions have a radical scavenging function and produce low oxidative species such as NO^•^_3_ and NO^•^_2_ (Eqs. (9) and (10))^84^.

17 $${{\text{NO}}}_{3}^{-}+ {{\text{OH}}}^{\cdot }\to {{\text{NO}}}_{3}^{\cdot }+{{\text{OH}}}^{-}$$

18 $${{\text{NO}}}_{3}^{\cdot }+ {{\text{H}}}_{2}\mathrm{O }+ {{\text{e}}}_{{\text{aq}}}^{-} \to {{\text{NO}}}_{2}^{\cdot }+{2{\text{OH}}}^{-}$$

Moreover, similar scavenging effects are due to the high proclivity of chlorine ions to combine with ^•^OH radicals and positive holes on TiO_2_/NP surface lead to generating lower reactive species (Eqs. (10)–(17)). On the other hand, the hindrance of TC molecule accessibility to ^•^OH radicals due to the competition of pollutant molecules with these ions for reaction with ^•^OH radicals may be a contributing cause for the declined efficacy observed in the presence of chloride^85^.

19 $${{\text{Cl}}}^{-}+ {{\text{h}}}^{+}\to {{\text{Cl}}}^{\cdot }$$

20 $${{\text{Cl}}}^{-}+ \cdot {\text{OH}}\to {{\text{Cl}}}^{\cdot }+{{\text{OH}}}^{-}$$

21 $${{\text{Cl}}}^{-}+ \cdot {\text{OH}}\to {{\text{ClOH}}}^{\cdot -}$$

22 $${{\text{ClOH}}}^{-}+{{\text{H}}}^{+} \to {{\text{Cl}}}^{\cdot }+{{\text{H}}}_{2}{\text{O}}$$

23 $${{\text{Cl}}}^{\cdot }+{{\text{Cl}}}^{-}\to {{\text{Cl}}}_{2}^{\cdot -}$$

24 $${{\text{Cl}}}_{2}^{\cdot -}+{{\text{Cl}}}_{2}^{\cdot -}\to {{\text{Cl}}}_{2}+{2{\text{Cl}}}^{-}$$

25 $${{\text{Cl}}}^{\cdot }+{{\text{Cl}}}^{\cdot } \to {{\text{Cl}}}_{2}$$

26 $${{\text{Cl}}}_{2}+{{\text{H}}}_{2}\mathrm{O }\to {\text{HOCl}}+{\text{HCl}}$$

27 $$\mathrm{HOCl }\to {{\text{H}}}^{+}+{{\text{OCl}}}^{-}$$

The findings indicate that the existence of sulfate ions resulted in the least inhibitory effect, possibly due to the interaction with active species and subsequent formation of SO_4_^•^ radicals possessing a greater redox potential (2.5–3.1 eV) than ^•^OH radicals (Eqs. (19), (20))^85^.

28 $${\mathbf{S}\mathbf{O}}_{4}^{2-}+{\mathbf{h}}^{+}\to {\cdot \mathbf{S}\mathbf{O}}_{4}^{2-}$$

29 $${{\text{SO}}}_{4}^{2-}+\cdot {\text{OH}}\to {\cdot {\text{SO}}}_{4}^{2-}+{{\text{OH}}}^{-}$$

### Mineralization and reusability of TiO_2_/pyrite

**Figure 10:**
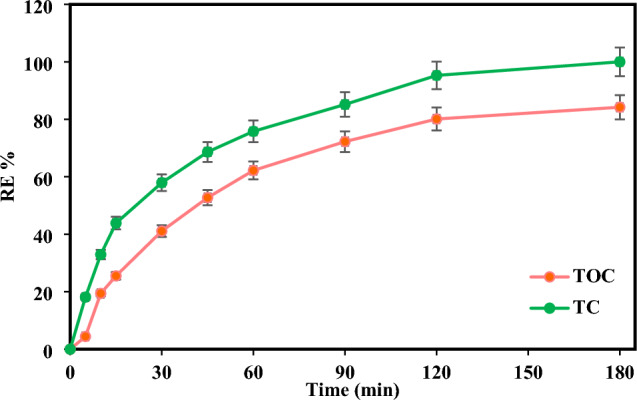
Removal efficiency of TOC by TiO_2_/NP at optimum conditions ([TC] = 30 mg/L, pH = 7, catalyst doe = 2 g/L and light intensity = 60 mW/cm^2^).

Complete removal of TC does not mean that mineralization has been completed, and CO_2_ and H_2_O have been produced, so in this work, the mineralization of TC by the TiO_2_/NP system was investigated by measuring TOC elimination. Figure 10 demonstrates that, considering optimal circumstances, TC was entirely eliminated within 180 min, while TOC removal reached 84%. This suggests that the entire mineralization of TC during the TiO_2_/NP photocatalytic process required longer reaction times compared to the photocatalytic removal of TC. This phenomenon may arise as a result of the manufacturing of diverse products and their competing with TC molecules^61,80^.

**Figure 11:**
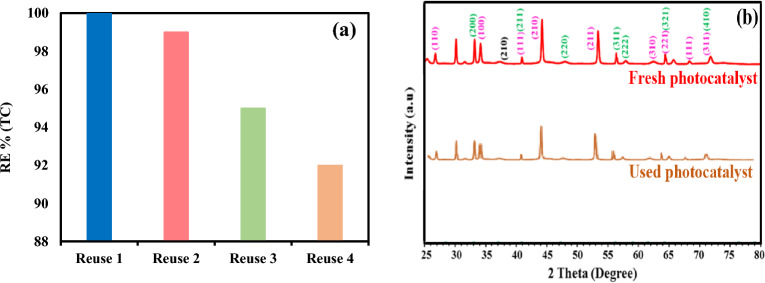
(**a**) the reusability of TiO_2_/NP at optimum conditions ([TC] = 30 mg/L, pH = 7, catalyst doe = 2 g/L and light intensity = 60 mW/cm^2^) (**b**) XRD pattern of fresh and used photocatalyst.

The durability and reusability of the catalyst are crucial variables in selecting and application of the catalyst for full scale. Herein, the reusability of TiO_2_/NP nanocomposite was examined during fourth repetitive runs under optimum conditions. By completing each run, the catalyst was isolated from the solution, rinsed with water, and subsequently stored at 90 °C for 4 h for the subsequent run. As depicted in Fig. 11a, only an 8% decrease in the efficiency of TC removal occurred following four cycles of catalyst utilization. Therefore, it indicated the nanocomposites high reusability and good photocatalytic activity. Nonetheless, the slight decrease in elimination effectiveness may be ascribed to the by-product formation during the elimination process, which could accumulate on the nanocomposite surface and obstruct the active sites on the catalyst surface, thereby hindering the removal of TC^86^. Furthermore, the photocatalytic efficiency of TiO_2_/NP probably declined due to the nanocomposite surface passivation and the number of ferrous ions released from the pyrite, which occurred after multiple uses of the catalyst^87^. Moreover, XRD analysis was used to further investigate the stability of the photocatalyst. The XRD pattern (Fig. 11b) indicated that the XRD peaks of the fresh and used photocatalyst are relatively constant, with a slight decrease in peak intensity due to minimal Fe leaching. The results indicated that the release of Fe was 0.134 mg/L after 4 cycles, confirming the high physicochemical stability of the photocatalyst, as the leaching of metals was less than 0.25 mg/L, demonstrating the proper stability of the photocatalyst^88^.

### Possible tetracycline photocatalytic degradation pathways

**Figure 12:**
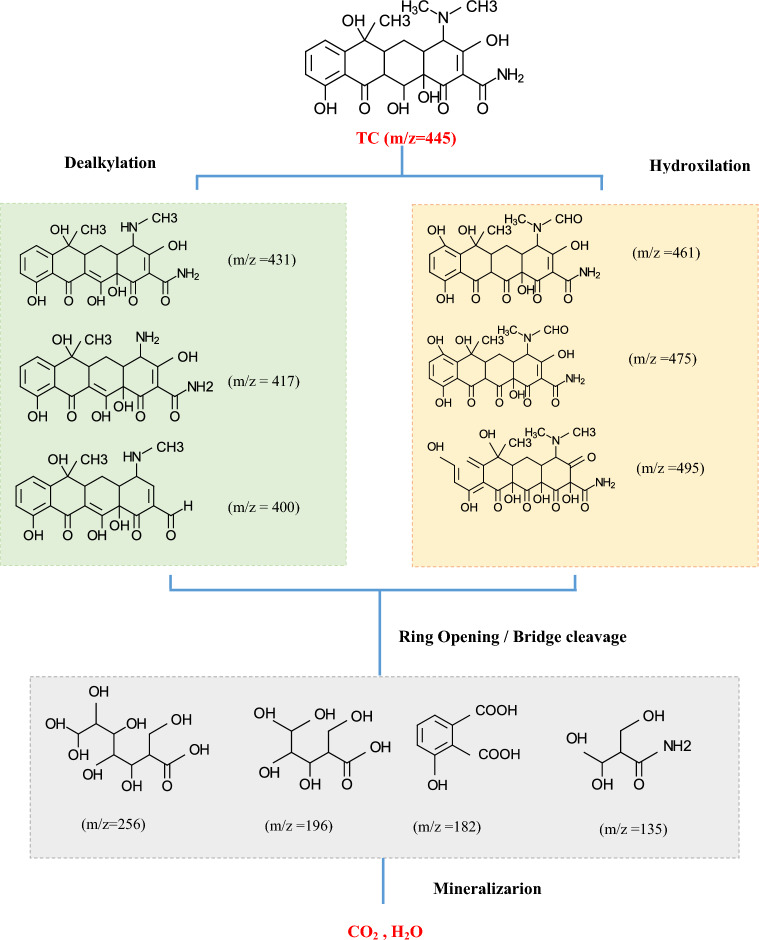
Feasible photocatalytic degradation pathways of TC in TiO_2_/NP system.

The photocatalytic breakdown of TC varies according to the type of photocatalyst and reaction conditions, leading to the formation of diverse by-products. Many studies showed that TC is generally converted into short-chain compounds and finally H_2_O and CO_2_ under various chemical processes such as dealkylation, hydroxylation, and ring opening^89,90^. The proposed pathway of TC decomposition in present work (Fig. 12) is based on the pathways proposed in previous studies related to the photocatalytic decomposition of TC in TiO_2_-based systems.

In one pathway, TC undergoes various reactions such as dealkylation and demethylation, as a result, some intermediates may be generated due to the elimination of functional groups with low binding energy such as dimethylamine^23,80^. Many studies have shown that in this pathway an intermediate with m/z 431 is generated, subsequently other intermediates with m/z 417, 400, 373, 339 are produced^91,92^. Further oxidation in the attendance of ROS including ·OH, ·O_2_^−^ and h^+^, leads to the generation of smaller intermediates through ring opening and central carbon cleavage reactions. These byproducts can be more oxidized to produce tiny organic molecules including organic acids, alcohols, H_2_O, and CO_2_^93,94^.

In another pathway, the attendance of a weak π bond in the TC structure facilitates the addition of hydroxyl groups through a hydroxylation process. This leads to the origination of the main intermediate with m/z = 461^95^. Further, by adding more hydroxyl groups, the mass-to-charge ratio increases, and other intermediates with m/z 475 and 495 are produced^80^. The addition of hydroxyl groups in the structure of TC causes the double bond in its structure to be destroyed, subsequently, with the attack of more oxidant species, intermediates with a lower number of rings with m/z 256, 196, 182, 135 are generated^91,92,96,97^. Generally, during the hydroxylation process and after adding OH in the tetracycline structure through oxidation and mineralization mechanisms, the aromatic ring undergoes breakage, resulting in the establishment of intermediates, as well as the production of CO_2_, H_2_O, and inorganic ions.

## Conclusion

The current work looked into the photocatalytic effectiveness of synthesized TiO_2_/NP for the decomposing of TC under visible light exposure. The findings suggest that the prepared nanocomposite demonstrated effective performance in removing TC from the aqueous solution., so that 100% of TC was removed under optimal conditions within 180 min reaction time. However, the photocatalytic activity of catalyst could be changed by variation in experimental conditions. The removal rate was accelerated by increased light intensity and the addition of optimum TiO_2_/NP dosage. Nevertheless, subsequent increases in the dosage of nanocomposite reduced the photocatalytic efficacy due to the agglomeration of catalysts and shortage of active sites to adsorb pollutants. Furthermore, photocatalytic process had lower efficiency by increasing the TC concentration. The predominant reactive oxidation species identified by adding quenching agents were hydroxyl radicals, which were crucial for photocatalytic activity. Also, reusability experiments of the TiO_2_/NP revealed that after four cycles, the photocatalytic efficiency does not significantly decline. Generally, it can be concluded that the TiO_2_/NP system had a suitable efficiency under optimized experimental conditions for TC removal. However, since the present investigation was conducted at a lab scale on synthetic effluent, the photocatalytic efficiency may differ from actual wastewater. Therefore, further studies are suggested to evaluate this photocatalytic process at a pilot scale using wastewater effluent.

## Data Availability

All data generated or analysed during this study are included in this published article.
